# Risk Neutral Measure Determination from Price Ranges: Single Period Market Models

**DOI:** 10.3390/e20070508

**Published:** 2018-07-04

**Authors:** Henryk Gzyl, German Molina, Enrique ter Horst

**Affiliations:** 1 Centro de Finanzas, IESA, Caracas 1011, Venezuela; 2 Idalion Capital Group, Quantitative Trading, 12 Hay Hill, London W1J 8NR, UK; 3 University of the Andes School of Management (Uniandes), Cl. 21 #1-20, Edificio SD, Bogotá, Colombia

**Keywords:** risk neutral measures, maximum entropy with errors in the data, bid-ask prices

## Abstract

Risk neutral measures are defined such that the basic random assets in a portfolio are martingales. Hence, when the market model is complete, valuation of other financial instruments is a relatively straightforward task when those basic random assets constitute their underlying asset. To determine the risk neutral measure, it is assumed that the current prices of the basic assets are known exactly. However, oftentimes all we know about the current price, or that of a derivative having it as underlying, is a bid-ask range. The question then arises as to how to determine the risk neutral measure from that information. We may want to determine risk neutral measures from that information to use it, for example, to price other derivatives on the same asset. In this paper we propose an extended version of the maximum entropy method to carry out that task. This approach provides a novel solution to this problem, which is computationally simple and fast.

## 1. Preliminaries and Problem Statement

Consider a one period (static) market model, in which there is a riskless asset, with prices $S_{0}\left(0\right)$ and $S_{0}\left(1\right)=\left(1+r\right)S_{0}\left(0\right),$ (or, alternatively, $S\left(1\right)=e^{r}S\left(0\right)$ when continuous time composition of returns is used), and *K* risky assets, with prices $S_{k}\left(0\right)$ and $S_{k}\left(1\right)$, which are modeled as random variables in some appropriate probability space (Ω,F,P). The probability measure *P* is used for forecasting or risk valuation purposes. However, to compute the price of financial assets in this market one should use a probability measure *Q*, with respect to which the market is a fair casino. Such measures are called risk neutral measures and are characterized by the property

(1) $$E_{Q}\left[\frac{S_{k}\left(1\right)}{1+r}\right]=S_{k}\left(0\right)\;\;\;\left(\mathrm{or}\;\;\;E_{Q}\left[e^{-r}S_{k}\left(1\right)\right]=S_{k}\left(0\right)\right)\;\;k=1,\ldots ,K.$$

The notation $E_{P},E_{Q}$ denotes expectation with respect to the probability laws *P* and *Q* [1,2]. This characterization can be thought of as a system of equations from which *Q* can be determined. First, we must determine the risk measure. If $F(S_{1}\left(1\right),\ldots ,S_{K}\left(1\right))$ denotes the payoff of some derivative at time t=1, then the market is complete. Therefore, when there is only one risk neutral measure, the price of the derivative is given by

(2) $$\pi \left(F\right)=E_{Q}\left[\frac{F(S_{1}\left(1\right),\ldots ,S_{K}\left(1\right))}{1+r}\right].$$

This last statement points to several related problems. If risk neutral measures are not unique, how can they be determined? In addition, what happens with derivatives on such assets? Furthermore, to further define the focus of our contribution, if all that is known is the market price of a few derivatives having the assets as underlying, can (2) be used to determine *Q* and price other derivatives?

If the whole class M of risk neutral measures is known, the bid-ask range for the price of an asset is given by

$$bid\left(S_{k}\right)=\inf\left\{E_{Q}\left[\frac{S_{k}\left(1\right)}{1+r}\right]:Q\in M\right\}\;\;ask\left(S_{k}\right)=\sup\left\{E_{Q}\left[\frac{S_{k}\left(1\right)}{1+r}\right]:Q\in M\right\}.$$

If a unique risk neutral measure exists, oftentimes current prices $S_{k}\left(0\right)$ are only known to lie within a bid-ask range, or the information about the prices is to be determined from the price of derivatives, usually called vanilla option prices, known up to a price range. This is more prevalent for the more illiquid assets, where trades may occur more seldom and the prevailing bid and ask levels provide most of the information available about the asset price.

The existence of the range may simply show the liquidity forces in a standard bid/ask spread, but it could also reflect the fact that it may be impossible to value the asset, and all the market-maker may propose is a price range, which can be arbitrarily large to reflect the market-maker’s uncertainty, skewness, and/or risk aversion [3]. Examples of this line of work include (1) methods for determination of bounds on option prices [4]; (2) direct analytical bounds proposed by [5]; (3) static (one period market models) arbitrage bounds, such as those in [6,7], using linear programming methods; (4) bounds using comonotonicity and copula arguments, such as those in [8,9]; or (5) more recent mass transportation arguments to obtain bounds, such as those proposed by [10].

The mid price is oftentimes assumed to be the actual price. However, market makers may show skewness in their preferences when pricing. Additionally, bid and ask volumes offered may reflect an imbalance between buyers and sellers.

Thus, when prices are only known up to a range, call it $\left[b_{k},a_{k}\right]$ for k=1,…,K, then the problem becomes:

(3) $$\mathrm{Determine}\;a\;\mathrm{probability}\;Q\;\mathrm{such}\;\mathrm{that}\;E_{Q}\left[\frac{S_{k}\left(1\right)}{1+r}\right]\in \left[b_{k},a_{k}\right]\;\;k=1,\ldots ,K$$

where $b_{k}$ and $a_{k}$ represent the bid and ask prices, respectively, for asset *k*.

Continuous time compounding convention may be used, and there may be more than one risk free rate available in the market. Thus, if $S\left(1\right)=S\left(0\right)e^{\rho }$ is used to model the future price, in which ρ denotes the logarithmic rate of return of the random asset, problem (3) becomes:

(4) $$\mathrm{Determine}\;a\;\mathrm{probability}\;Q\;\mathrm{such}\;\mathrm{that}\;E_{Q}\left[e^{\rho_{k}}\right]\in \left[e^{r_{m}},e^{r_{M}}\right]\;\;k=1,\ldots ,K.$$

We denote $r_{m}$ and $r_{M}$ as the minimum and maximum risk free rates of return, respectively. A problem that has been explored in the literature consists of determining the risk neutral probability from the knowledge of the prices of a few options with different strike prices. Let ${\left(\cdot \right)}^{+}$ denote the maximum between zero and the argument therein. This time our problem could be restated as

(5) $$\mathrm{Determine}\;a\;\mathrm{probability}\;Q\;\mathrm{such}\;\mathrm{that}\;E_{Q}\left[{\left(S\left(1\right)-K_{k}\right)}^{+}\right]\in \left[b_{k},a_{k}\right]\;\;k=1,\ldots ,K.$$

As reflected in (5), we may only know the price of a few call options (calls, puts, or both) up to a range. Again, consider the model $S\left(1\right)=S\left(0\right)e^{\rho }.$ If S(0) is only known up to a range, suppose that S(0) is the mid point of the bid-ask interval, even though we know that is not the correct price, and rephrase the problem as

(6) $$\mathrm{Determine}\;a\;\mathrm{probability}\;Q\;\mathrm{such}\;\mathrm{that}\;E_{Q}\left[S\left(0\right)e^{\rho }\right]+\epsilon =S\left(0\right)e^{r}.$$

where ϵ is the mispricing to be determined along with the determination of the pricing measure Q. To invoke a physical analogy, $S\left(0\right)e^{r}$ is the result of a measurement contaminated by errors, which on average equals ϵ. The first term in the left hand side of (6) is the “true” price, while the second term is the estimator of the mispricing which has to be determined, as well.

Note that when the results for the single period market are taken together with an assumption of independence of the increments of the prices, then the results can be easily made part of a dynamical problem.

The use of maximum entropy based methods to obtain risk neutral measures for asset pricing, that is, to solve inverse problems like (1) or (5) with exact data is not new. This approach has been explored in [11,12,13,14,15,16,17,18]. among others. However, as previously outlined, our use of the maximum entropy method differs from these, in that we expand the problem to consider the datum to be a price range instead of a given price, an assumption more aligned with some real life applications. The way in which maxentropic methods fit in the context of a standing effort to determine risk neutral measures from option prices, especially in the context of the binomial model, was the subject of an extensive review by [19], as well as [20]. The latter use maximum entropy methods to determine a pricing measure within the scope of range data. However, they deal with the price ranges differently. Other approaches include the use of a different entropy functional, as in [21], as well as work on the determination of risk neutral densities using approximations by rational functions, as in [22].

Our approach to solve these inverse problems (3)–(6) involves the extension of the maximum entropy method to incorporate errors in the data, as proposed in [23]. Miyahara [24] discusses the role that the method of maximum entropy plays and its relationship to the Esscher transform plays as an important role for valuation in incomplete markets. Finally, Zhou et al. [25] provide a more extensive review of the recent literature. Besides the possibility of handling errors and data in ranges, an important feature of the maximum entropy based methodology is its model-independent, parameter-free nature, which relies solely on the available market data as input.

The remainder of this paper is organized as follows. In Section 2, we recall the basics of the maximum entropy technique in several stages. First, we introduce the standard maximum entropy method in Section 2.1. This serves as a stepping stone for the second method, covered in Section 2.2. For the extension necessary to solve problem (6), we provide an extension, which is covered in Section 2.3. Finally, in Section 3 we examine some examples that cover the problems stated above. We end with some concluding remarks.

## 2. The Method of Maximum Entropy with Errors in the Data

### 2.1. The Standard Method of Maximum Entropy

The historical antecedents of the standard method go back to [26], whose proposal is now called *tilting* in the statistical literature, as well as [27], who extended the work by Gibbs at the turn of the 20th century on the foundations of statistical physics. In [27], the generic problem consisting of determining a probability density from the knowledge of the expected values of a few random variables was formulated as a variational problem. It is the method proposed by Jaynes that shall be used as a stepping stone for the proposed ways to solve (3) and (6). A related procedure was discussed by [28], and later on by [29], motivated by applications in statistics. In the rest of this preamble we shall state the solution of the standard maximum entropy (*maxent* for short) procedure.

Consider the generic problem consisting of finding a density *g*, with respect to a probability measure *P*, defined on a measurable space (Ω,F), and satisfying the integral constraint

(7) $$E_{P}\left[gX_{i}\right]=\int_{\Omega }gX_{i}dP=m_{i},\;\;i=1,\ldots K.$$

$X_{i}:\Omega \to R$ are given random variables, and *g* is the density of the risk neutral measure *Q* with respect to P. In relation to (1), note that $X_{i}=S_{i}\left(1\right)/\left(1+r\right)$ and $m_{i}=S_{i}\left(0\right).$ The variational method proposed by Jaynes consists in defining a concave function (the entropy) on the class of densities satisfying the constraints and choosing the one that maximizes it. The entropy function is defined by

(8) $$S_{P}\left(Q\right)=S_{P}\left(g\right)=\left\{\begin{array}{c} -\int_{\Omega }g\ln gdP\\ -\infty \;\mathrm{if}\;\mathrm{the}\;\mathrm{integral}\;\mathrm{is}\;\mathrm{not}\;\mathrm{defined}. \end{array}\right.$$

It is well known (see [27]) or the work by [30,31] in which mathematical nuances are explained), that the entropy maximization problem, consisting in finding a density $g^{*}$ that solves the problem

(9) $$\mathrm{Find}\;\;g^{*}=\underset{g}{arg sup}\left\{S_{P}\left(g\right)\;|\;g\in D\left(m\right)\right\},$$

where m is the k-vector with components $m_{i},\;i=1,\ldots K$ and D(m) denotes the class of densities satisfying (7), has a unique solution given by

(10) $$g^{*}=\frac{e^{-\sum_{i=1}^{K}\lambda_{i}^{*}X_{i}}}{Z(\lambda^{*})}.$$

In (10) the normalization factor Z(λ) is defined by the obvious

$$Z\left(\lambda \right)=\int_{\Omega }e^{-\sum_{i=1}^{K}\lambda_{i}X_{i}}dP.$$

This function is well defined on $\left\{\lambda \in R^{K}|Z\left(\lambda \right)<\infty \right\}$ and Z(λ) is convex on that (convex) set. In (10), $\lambda^{*}$ is to be determined minimizing the strictly convex function

(11) Σ(λ,m)=ln(Z(λ))+〈λ,m〉.

Building on convex duality theory (see Theorem 3.3.5 in [30]), the maximization/minimization procedures are such that

$$\sup\left\{S\left(g\right)|g\in D\left(m\right)\right\}=\inf\left\{\Sigma \left(\lambda ,m\right)|\lambda \in R^{M}\right\},$$

which is equivalent to

(12) $$S_{P}\left(g^{*}\right)=\ln\left(Z\left(\lambda^{*}\right)\right)+\langle \lambda^{*},m\rangle$$

since, in our problem, Σ(λ,m) is finite and strictly convex on $R^{M}.$

### 2.2. Nested Sequence of Entropy Maximization Problems

Using the generic notation of the previous section, instead of (7), consider the following extended version of it: Determine a density *g* such that

(13) $$E_{P}\left[gX_{i}\right]=\int_{\Omega }gX_{i}dP\in \left[b_{i},a_{i}\right]\;\;i=1,\ldots K.$$

Let us denote by D the class of densities satisfying (13). To simplify the notation, define $C=\prod_{i=1}^{K}\left[b_{i},a_{i}\right].$ Then notice that

(14) $$\sup\left\{S_{P}\left(g\right)\;|\;g\in D\right\}=\sup\left\{\sup\left\{S_{P}\left(g\right)\;|\;,g\in D\left(m\right)\right\}\;|\;m\in C\right\}$$

If we now invoke (12), the last identity can be replaced by

$$\sup\left\{S_{P}\left(g\right)\;|\;g\in D\right\}=\sup\left\{\inf\left\{\Sigma \left(\lambda ,m\right)|\lambda \in R^{K}\right\}\;|\;m\in C\right\}$$

Observe that the dependence of Σ(λ,m) on both λ and *m* is differentiable, and the joint derivative is positive, thus the maximization and minimization can be interchanged to obtain

$$\sup\left\{S_{P}\left(g\right)\;|\;g\in D\right\}=\inf\left\{\sup\left\{\Sigma \left(\lambda ,m\right)|\;m\in C\right\}\;|\;\lambda \in R^{K}\right\}.$$

Now, due to the special form of Σ(λ,c), it suffices to compute sup{〈λ,m〉|m∈C}, making use of the simple-to-verify equality $\sup\left\{\langle \lambda ,y\rangle |y\in {\left[-1,1\right]}^{K}\right\}={∥\lambda ∥}_{1}.$

Consider the affine mapping T(m)=Dm+h, where D is diagonal with elements $2/(b_{k}-a_{k})$ and $h_{k}=-\left(a_{k}+b_{k}\right)/\left(a_{k}-b_{k}\right).$ This maps $\left[b_{1},a_{1}\right]\times \ldots \times \left[b_{K},a_{K}\right]$ bijectively onto ${\left[-1,1\right]}^{K}.$ Then, if we define

$$\delta_{C}^{*}\left(\lambda \right)\equiv \sup\left\{\langle \lambda ,m\rangle |m\in C\right\}={∥D^{-1}\lambda ∥}_{1}-\langle D^{-1}\lambda ,h\rangle .$$

with

$$∥D^{-1}{\lambda ∥}_{1}=\sum_{i=1}^{K}\frac{\left(a_{i}-b_{i}\right)\left|\lambda_{i}\right|}{2}\;\;\mathrm{and}\;\;<D^{-1}\lambda ,h>=-\sum_{i=1}^{K}\frac{\left(b_{i}+a_{i}\right)\lambda_{i}}{2}.$$

As a first step towards a solution, we have the following:

**Lemma** **1.** *With the notations introduced above, set*

(15) $$\Sigma \left(\lambda \right)=\ln\left(Z\left(\lambda \right)\right)+\delta_{C}^{*}\left(\lambda \right)$$

*Then Σ(λ) is strictly convex in λ.*

The proof of these assertions is simple. On one hand, a simple application of Hölder’s inequality yields the strong convexity of lnZ, and the strong convexity of $\delta_{C}^{*}.$ is apparent from its specification just above the statement of the lemma.

Observe that $\partial \delta_{C}^{*}\left(\lambda \right)/\partial \lambda_{i}$ is defined except at $\lambda_{i}=0,$ where it is sub-differentiable (see [30]). More explicitly,

$$\begin{array}{c} \partial \delta_{C}^{*}\left(\lambda \right)/\partial \lambda_{k}=a_{k}\;\;\mathrm{when}\;\;\lambda_{k}>0,\\ \partial \delta_{C}^{*}\left(\lambda \right)/\partial \lambda_{k}=b_{k}\;\;\mathrm{when}\;\;\lambda_{k}<0,\\ \partial \delta_{C}^{*}\left(\lambda \right)/\partial \lambda_{k}\in \left(b_{k},a_{k}\right)\;\;\mathrm{when}\;\;\lambda_{k}=0 \end{array}$$

And to close down, we have the general, generic statement:

**Theorem** **1.** Suppose that the infimum of Σ(λ) is reached at some $\lambda^{*}$ in the interior of $\{\lambda \in R^{K}|Z\left(\lambda \right)<\infty \}.$ Then the solution to the maximum entropy problem with constraints (13) is

(16) $$g^{*}=\frac{1}{Z(\lambda^{*})}e^{-\sum_{k=1}^{K}\lambda_{k}^{*}X_{k}}.$$

*Due to the computation in (16), it is clear that*

$$\int_{\Omega }g^{*}X_{k}dP\in \left[b_{k},a_{k}\right],\;\;for\;\;k=1,\ldots ,K.$$

Implicit in the above statement is the fact that the interior domain of the function non-empty, that the function is bounded below, and that the minimum is not reached at the boundary of the domain. The proof of the result is simple. Since by assumption the minimum is reached at an interior point, we can differentiate Σ(λ). The first order condition for $\lambda^{*}$ yields the result by simple inspection. In this way we obtain the simplest possible version of the more general case discussed in Section 2.2 and in Proposition 3.1.5 in [30], according to which $\lambda^{*}$ is a minimizer if and only of the condition 0∈∂Σ(λ), which we assumed to hold in the statement of the theorem. In addition, the computation above the statement of the theorem makes the subgradient explicit. This computation is essential for the numerical procedures.

### 2.3. Maxentropic Density Reconstruction from Data with Errors

In this section we will deal with the problem of determining a density *g* such that dQ=gdP satisfies the following extended version of (7):

(17) $$E_{Q}\left[X_{i}\right]+\epsilon_{i}=E_{P}\left[gX_{i}\right]+\epsilon_{i}=m_{i},\;\;i=1,\ldots K$$

where again $X_{i}$:Ω→R are given random variables, and $\epsilon_{i}$ are interpreted as errors in the measurement process of $m_{i}.$ In the examples treated below, we shall think of the $\mu_{i}$ as the prices observed up to possible mispricing: $E_{P}\left[gX_{i}\right]$ will be the true prices, and $\epsilon_{i}$ will measure the amount of mispricing. A source of mispricing might occur when the true price is calculated numerically, or if the price of the asset is quoted up to the wrong decimal figure. For simplicity (although this assumption can easily be relaxed at the expense of encumbering the notation), we suppose that all errors fall within the same range [−c,c], and that the (unobserved) error is to be modeled as the expected values of random variables $\eta_{i}$ taking values in [−c,c], that is

(18) $$\epsilon_{i}=E_{Q_{n}}\left[\eta_{i}\right].$$

As the right hand side and the first term on the left hand side of (17) are averages over observations, so is the error term, and to estimate the error, it suffices to determine the measure $Q_{n}$ (the subscript *n* stands for “noise”). Note as well that if $m_{i}$ is the “noisy” price, and $\epsilon_{i}$ is the amount of mispricing, it makes sense to refer to $m_{i}-\epsilon_{i}=E_{Q}\left[X_{i}\right]$ as the “correct” price.

The setup of the first section is augmented as follows: Our sample space will be $\Omega \times {\left[-c,c\right]}^{K}$ with $F⊗B({\left[-c,c\right]}^{K}).$ As reference measure we take $dP⊗\prod_{i=1}^{K}dP_{n}\left(\eta_{i}\right).$ Here $\eta_{i}$ stands for the coordinate on the i-th copy of [−c,c]. We set $dP_{n}\left(\eta_{i}\right)=\left(\delta_{-c}\left(d\eta_{i}\right)+\delta_{c}\left(d\eta_{i}\right)\right)/2.$, with $\delta_{a}\left(d\eta \right)$ being the Dirac point mass at the point a. Additionally, the density of any probability $dQ_{n}$ with respect to $dP_{n}$ is given by a parameter q, that is $dQ\left(\eta_{i}\right)=q\delta_{-c}\left(d\eta_{i}\right)+\left(1-q\right)\delta_{c}\left(d\eta_{i}\right).$ This presentation of $Q_{n}$ renders it normalized at the outset.

Having introduced all necessary notation, we can translate the results from the first section to this case. The entropy to be minimized has the following form:

$$S\left(g,\alpha \right)=-\int_{\Omega }g\left(x\right)dP\left(x\right)-\sum_{i=1}^{K}\left(q_{i}\ln p_{i}+\left(1-q_{i}\right)\ln\left(1-q_{i}\right)\right).$$

This time the densities that minimize the entropy are

(19) $$\begin{array}{cc} g^{*}\left(y\right) & =\frac{e^{-\sum_{k=1}^{M}\lambda_{k}^{*}y^{\alpha_{k}}}}{Z(\lambda^{*})}\\ q_{i} & =\frac{e^{c\lambda_{k}^{*}}}{e^{c\lambda_{k}^{*}}+e^{-c\lambda_{k}^{*}}}. \end{array}$$

The normalization factor Z(λ) is defined by the obvious

$$Z\left(\lambda \right)=\int_{\Omega }e^{-\sum_{i=1}^{K}\lambda_{i}X_{i}}dP,$$

and $\lambda^{*}$ is determined minimizing the strictly convex (dual entropy) function

(20) $$\Sigma \left(\lambda \right)=\ln Z\left(\lambda \right)+\sum_{k=1}^{M}\ln\left(e^{c\lambda_{k}}+e^{-c\lambda_{k}}\right)+<\lambda ,m>.$$

Once $g^{*}$ and $q_{i}$ are at hand, the true value of the $X_{i}$ is given by

$$\int_{\Omega }x_{i}g^{*}\left(x\right)dP\left(x\right)$$

and the (estimated) measurement error $\epsilon_{i}$ is given by

$$\epsilon_{i}=-cq_{i}+c\left(1-q_{i}\right).$$

Note that these two numbers satisfy (17).

## 3. Numerical Examples

The core of the numerical examples described below is the minimization of the function Σ(λ). The numerical algorithm is a combination of the Newton-Raphson method with the Barzilai-Borwein step reduction procedure, already implemented in the R library by [32]. This step reduction procedure is important because in our examples the functions to be minimized are very flat near the minimum. In the examples we consider, the interior of $\left\{\lambda \in R^{K}|Z\left(\lambda \right)<\infty \right\}$ is non-empty and Σ(λ) has a minimum there.

To finish the preamble, implicit in the statement of the problems is that the constraints are feasible. An easy and practical test of infeasibility is the non-existence of the minimizer.

### 3.1. Risk Neutral Prices from Option Prices: Discrete Case

Consider the following simple example, taken from the monograph by [33], because of its potential use for scenario analysis. Here we change the original market data, listed in Table 1, into data provided as bid-ask prices as in Table 2. We thus have an example in which the direct determination of the risk neutral probability by solving a linear system is impossible. Suppose that the price range of the Spanish equity index (*indice Bursátil Español*, literally Spanish Exchange Index), or in short IBEX, is split into six tranches, each of which determine a market state. Table 3 summarizes this process.

As indicated in Table 3, there are six market states in this market model, i.e., $\Omega =\{\omega_{1},\ldots \omega_{6}\}.$ Certainly F=P is the class of all subsets of Ω and, given no other prior information, we may take $P(\omega_{k})=1/6.$ In this market, the investor has the following assets at hand: a quarterly treasury bond with a yearly yield of 12%, as well as five call options described as follows:

$$X_{k}\left(\omega_{j}\right)={\left(S\left(1\right)\left(\omega_{j}\right)-K_{k}\right)}^{+}$$

where S(1) denotes the value of the IBEX at maturity, ${\left(x\right)}^{+}=max\left(0,x\right)$, and its values at the six market states are the “levels” mentioned in Table 3.

It is a very simple exercise to verify that this market model is complete. We list the risk neutral probabilities in Table 4 below. However, the price data could only be available as ranges for the options, for example as in Table 2.

For this example, (13) becomes

$$\frac{1}{6}\sum_{j=1}^{6}g\left(\omega_{j}\right)X_{k}\left(\omega_{j}\right)\in \left[b_{k},a_{k}\right]\;\;k=1,\ldots 5.$$

Let us now make explicit the $X_{k}.$ In our example

$$X_{k}\left(\omega_{j}\right)={\left(S\left(1\right)\left(\omega_{j}\right)-K_{k}\right)}^{+}$$

where S(1) denotes the value of the IBEX at maturity, and its values at the six market states are the “levels” mentioned in Table 3.

In the present example, the function Z(λ) is given by

$$\frac{1}{6}\sum_{j=1}^{6}e^{-\sum_{i=1}^{5}\lambda_{i}X_{i}\left(\omega_{j}\right)}$$

which is part of the Σ(λ) appearing in (1).

If we apply the routine described at the end of Section 2, we obtain the risk neutral measure such that, if we compute the risk neutral prices of the observed options, we obtain Table 5, in which we list the prices and the quoted bid-ask intervals.

The pricing density $g^{*}\left(\omega_{j}\right)$ and the risk neutral probabilities $q\left(\omega_{j}\right)=g^{*}\left(\omega_{j}\right)/6$ listed in Table 4 are rounded to the fourth decimal place. In the last row, for comparison, we list the risk neutral probabilities (the Arrow-Debreu prices) of the market states.

**Figure 1:**
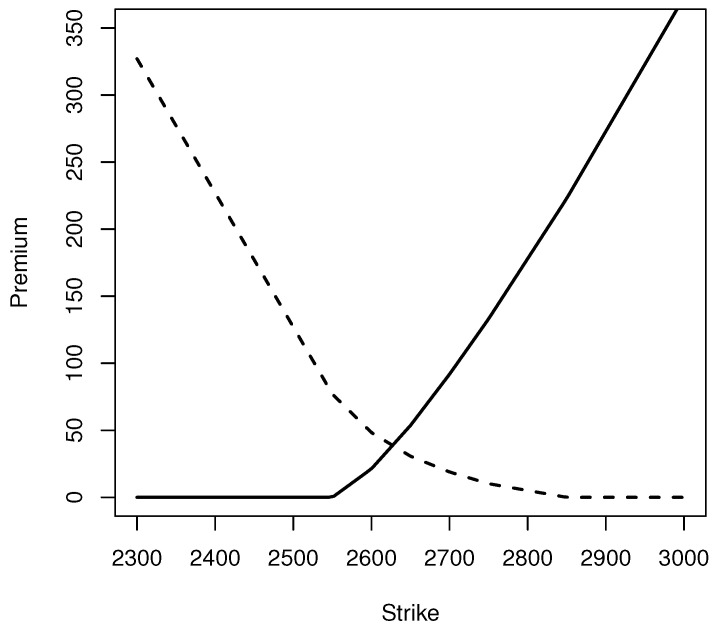
Call (continuous) and put (dashes) prices from maxentropic risk neutral measure.

From the reconstructed risk neutral probabilities, we obtain the European call and put price curves (K,C(K)) and (K,P(K)) displayed in Figure 1, alongside the prices computed from the risk neutral distribution computed from the exact prices. Note as well that C(K)−P(K)+K=2626.796 is the risk neutral price of the index.

### 3.2. Simple Continuous Example: The Risk Free Rate Is Uncertain

The next case consists of a twist on the basic Merton-Black-Scholes model. For this case, the computations can be performed analytically. The market model consists of a probability space (R,B(R),P), where *P* is the N(0,1) law. Suppose that we know that the future price S(1) of an asset is such that the logarithmic return is given by $\rho =\mu -\sigma^{2}/2+\sigma W,$ where W∼N(0,1). Notice that in this model $E_{P}\left[S\left(1\right)\right]=S\left(0\right)\exp\left(\mu \right).$

Suppose that in the market there exists a range $\left[r_{m},r_{M}\right]$ for the possible zero risk rates. In this context, in order to price assets that have *S* as underlying, we phrase the problem of determining a risk neutral measure as follows:

(21) $$\mathrm{Determine}\;a\;\mathrm{probability}\;Q<<P\;\mathrm{such}\;\mathrm{that}\;\;E_{Q}\left[\rho \right]\in \left[r_{m},r_{M}\right]-\sigma^{2}/2.$$

The reason for subtracting $\sigma^{2}/2$ is motivated by the last sentence of the first paragraph. It takes a few simple computations to note that

$$Z\left(\lambda \right)=E_{P}\left[e^{-\lambda \rho }\right]=e^{-\lambda \left(\mu -\sigma^{2}/2\right)+{\left(\lambda \sigma \right)}^{2}/2},$$

and that, for $r-\sigma^{2}/2\in \left[r_{m},r_{M}\right]-\sigma^{2}/2,$ there is one $\lambda^{*}$ such that

$$E_{P}\left[\rho \frac{e^{-\lambda \rho }}{Z(\lambda )}\right]=r-\sigma^{2}/2.$$

This leads to $\lambda^{*}=\left(r-\mu \right)/\sigma .$ Regarding $Q^{*}$, we obtain $E_{Q}\left(S\left(1\right)\right]=S\left(0\right)e^{r}.$ The value of the corresponding entropy is $S\left(Q^{*}\right)=-\lambda^{*2}/2.$ This solves the inner entropy maximization in (14).

The result of the outer maximization process depends on where μ lies, relative to $[r_{m},r_{M}].$ There are three cases to consider. We denote by $r^{*}$ the value at which the outer maximum is achieved. Clearly

(22) $$r^{*}=\left\{\begin{array}{c} \mu \;\;\mathrm{when}\;\;\mu \in [r_{m},r_{M}],\\ r_{m}\;\;\mathrm{when}\;\;\;\;\;\;\;\mu <r_{m}\\ r_{M}\;\;\mathrm{when}\;\;\;\;\;\;\mu >r_{M}. \end{array}\right.$$

The risk neutral asset prices that such maxentropic law provides is $S\left(0\right)E_{Q^{*}}\left[e^{X}\right]=S\left(0\right)e^{r^{*}}$.

### 3.3. Only the Bid-Ask Prices of the Asset Are Known

To establish some notation, recall that our motivation for problem (6) was the following: We are interested in finding a probability Q<<P such that $E_{Q}\left[S\left(1\right)\right]\in e^{r}\left[b,a\right].$ If we relate the future price of the asset to its current price by the model $S\left(1\right)=S\left(0\right)e^{\rho },$ a problem arises because we only know S(0) up to a range. To get around this difficulty, we set S(0)=(a+b)/2 and call it the observed price (just because *a* and *b* are observed) and assume that the true price is unknown. Then, besides determining a risk neutral measure, we want to determine the correct price, or equivalently, the amount of mispricing.

To cast the problem into the notation of Section 2.3, we consider the following market model: Set Ω=[0,∞) and FB([0,∞)), and, to finish, *P* is the distribution of $X=e^{\rho }$ with $\rho =\mu -\sigma^{2}/2+\sigma W$, and W∼N(0,1). As above $E_{P}\left[X\right]=e^{\mu }.$

Now, let c=(a−b)/(a+b), and consider the problem of determining a density *g* of a measure Q<<P and a number 0<q<1 such that

(23) $$\int_{0}^{\infty }xg\left(x\right)dP\left(x\right)+-cq+c\left(1-q\right)=e^{r}:=m$$

According to Section 2.3, in order to solve (23) we have to minimize (15) with Σ(λ,m) given by

$$\Sigma \left(\lambda ,m\right)=\ln\left(\int_{-\infty }^{\infty }\exp\left(-\lambda e^{\mu -\sigma^{2}/2+\sigma y}\right)\frac{e^{-y^{2}/2}}{\sqrt{2\pi }}dy\right)+\ln\left(e^{c\lambda }+e^{-c\lambda }\right)+\lambda m.$$

From this point onwards we have to proceed numerically. There is no problem with the convergence of the integral, but it does not lead to an analytic function at λ=0.

Once the minimizer $\lambda^{*}$ is at hand, we compute the “correct” price $\hat{S}\left(0\right)$ of the asset by

$$\hat{S}\left(0\right)=S\left(0\right)\frac{\int_{-\infty }^{\infty }e^{\mu -\sigma^{2}/2+\sigma y}\exp\left(-\lambda^{*}e^{\mu -\sigma^{2}/2+\sigma y}\right)\frac{e^{-y^{2}/2}}{\sqrt{2\pi }}dy}{\int_{-\infty }^{\infty }\exp\left(-\lambda^{*}e^{\mu -\sigma^{2}/2+\sigma y}\right)\frac{e^{-y^{2}/2}}{\sqrt{2\pi }}dy}$$

Now that we have estimated the correct price $\hat{S}\left(0\right),$ in order to plot the risk neutral density in terms of the price of the asset, we make the change of variables $x=\hat{S}\left(0\right)\exp\left(\mu -\sigma^{2}/2+\sigma y\right)$ and obtain

$$\frac{e^{-\lambda^{*}\left(x/\hat{S}\left(0\right)\right)}}{Z(\lambda^{*})}\frac{\exp\left(-\frac{1}{2\sigma^{2}}{\left(\ln\left(x/\hat{S}\left(0\right)\right)-\mu +\sigma^{2}/2\right)}^{2}\right)}{x\sigma \sqrt{2\pi }}$$

**Figure 2:**
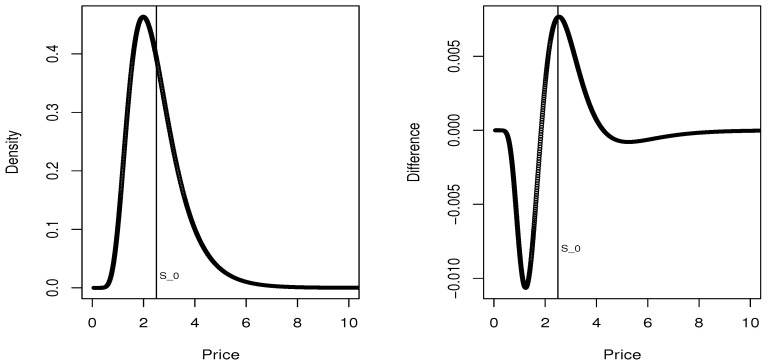
Maxentropic density and difference with true density.

For this problem, we considered μ=0.25, σ=0.40 and r=0.01. In addition, we considered S(0)=2.5 and, respectively b=S(0)(1−c) and a=S(0)(1+c) and values c=0.01, and c=0.02 and c=0.05 just to test the robustness of the method. In the left panel of Figure 2 we plot the maxentropic density, and in the right panel we plot the difference between the maxentropic density and the true risk neutral density for this example. The plot corresponds to the case c=0.01, which is already a high bid-ask spread.

### 3.4. Risk Neutral Measures from Option Prices

Here we consider an example discussed by [11,13,17,18]. Consider in particular the work by [11], in which the mathematical nuances of the problem are discussed.

The simplest version of the problem asks for determining the risk neutral measure of an asset of price S(1) if all that is known is that the prices of several options, say $\left(S\left(1\right)-K_{i}\right)$ fall in the ranges $[b_{i},a_{i}].$

As a market model we might consider (R,B(R),P) with *P* being the physical law of the underlying asset, which we suppose to be lognormal. That is, we suppose $S\left(1\right)=S\left(0\right)\exp\left(\mu -\sigma^{2}/2+\sigma W\right),$ as above, and we assume we know S(0), μ, σ, and the risk free rate r. For j=1,…J, let $X_{j}={\left(S\left(1\right)-K_{j}\right)}^{+}$ and for j=J+1,…M let $X_{j}={\left(K_{j}-S\left(1\right)\right)}^{+}$ be the cash flows at t=1 of a collection of call and put options of known strike prices $K_{1},\ldots K_{M}.$ Our aim this time is to find a measure *Q* having a density *g* with respect to *P* such that

$$E_{Q}\left[X_{j}\right]\in e^{r}\left[b_{j},a_{j}\right].$$

Since we do not know how prices are being assigned by the market makers, we proceed as in [13], and use the standard Black-Scholes methodology to price the options that we consider and then perturb the price around it to have the bid ask prices. That is, if we denote by p(K) and c(K) respectively, the prices of the European call and put options of strike price K, computed using the Black-Scholes formula, we set b=c(K)(1−f) and a=c(K)(1+f) as the bid-ask prices for the call, and b=p(K)(1−f) and a=p(K)(1+f), for the bid-ask prices for the put. We did the numerical work with f=0.075 as suggested by [13], but also tried a more realistic spread of f=0.01. The results were the same up to the 4-th decimal place.

For this example

$$Z\left(\lambda \right)=E_{P}\left[e^{-\sum_{j=1}^{M}\lambda_{j}X_{j}}\right]=\int_{0}^{\infty }e^{-h(\lambda ,y)}\frac{1}{y\sigma \sqrt{2\pi }}e^{-{\left(\ln y-\mu +\sigma^{2}/2\right)}^{2}/2\sigma^{2}}dy$$

where, to simplify notations, we set

$$h\left(\lambda ,y\right)=\sum_{j=1}^{J}\lambda_{j}{\left(S\left(0\right)y-K_{j}\right)}^{+}+\sum_{j=J+1}^{M}\lambda_{j}{\left(K_{j}-S\left(0\right)y\right)}^{+}.$$

Again, Z(λ) is well defined and log-convex for in the region $\lambda_{j}>0$ for 1≤j≤M. This is the input for the minimization of (15), after which we proceed as indicated in Theorem 1.

**Figure 3:**
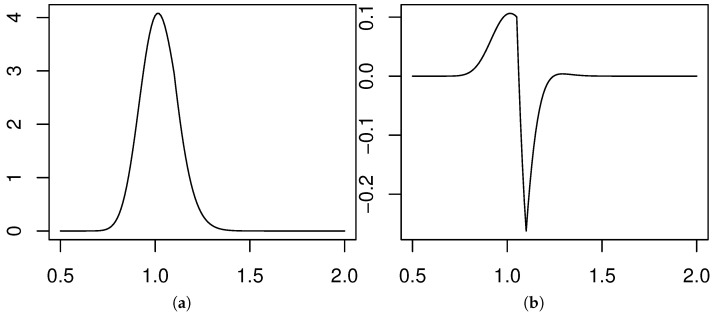
(**a**) Maxentropic risk neutral density from four price ranges. (**b**) Difference of reconstructions.

The numerical values of the parameters used were: μ=0.03, σ=0.1 and r=0.01. The current price of the asset was set at S(0)=1, and the two sets of strike prices that we used were the following: For M=4 we used {1.1,1.2} for the calls and {0.9,0.8} for puts, and for M=8 we considered {1.05,1.10,1.15,1.20} for the calls and {0.95,0.90,0.85,0.80} for the puts. The density obtained for M=4 is displayed in Figure 3a.

As the density obtained from eight moments looks very similar, we chose to plot the difference between the two densities in Figure 3b. Clearly the difference is extremely small.

**Figure 4:**
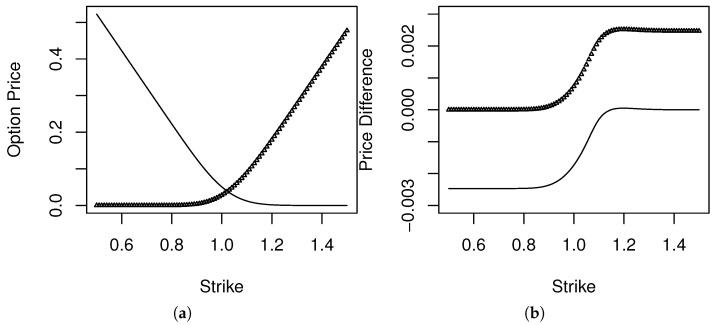
(**a**) Call (thick) and put (thin) prices computed with maxentropic density. (**b**) Differences in call and put prices.

Figure 4a presents the results of the computation of the prices of the call and put options performed with respect to the density determined by the maxentropic method using 4 option price ranges as inputs. The price of the call options is the increasing curve, and that of the puts is the decreasing curve.

We did the same with the maxentropic density reconstructed from eight price ranges. The difference between the prices obtained in each case is displayed in the right panel Figure 4b, using the same symbol as for the price curves in the left panel.

## 4. Concluding Remarks

The method of maximum entropy in the mean is a suitable technique to deal with linear inverse problems subject to convex constraints. The technique allows us to transform the problem of solving a system of integral equations, into a low dimensional convex optimization problem that can be easily solved numerically. In our case, the integral equations yield a risk neutral density from the prices of a few assets, and in our case the prices might be known up to a range.

Those risk neutral densities can then be used for pricing other derivatives that have the given assets as underlying. We mention that, even though the examples chosen align with those in the references cited, the method allows for any derivative having a given asset as underlying. However, as in the standard moment problem in statistics, the density obtained will reflect the information used as input.

## Figures and Tables

**Table 1:** Options data.

Strike Price	2550	2600	2650	2700	2750
Option price	73	47	31	20	10

**Table 2:** Bid-ask prices of the options.

Strike Price	2550	2600	2650	2700	2750
Option price	[69.2, 76.8]	[44.6, 49.4]	[29.4, 32.6]	[19, 21]	[9.5, 10.5]

**Table 3:** Market states for the IBEX data.

State	$\omega_{1}$	$\omega_{2}$	$\omega_{3}$	$\omega_{4}$	$\omega_{5}$	$\omega_{6}$
Level	2850	2750	2700	2650	2600	2550
Range	(2750,∞)	(2700,2750]	(2650,2700]	(2600,2650]	(2550,2600]	(−∞,2550]

**Table 4:** Risk neutral probabilities.

	$\omega_{1}$	$\omega_{2}$	$\omega_{3}$	$\omega_{4}$	$\omega_{5}$	$\omega_{6}$
$g^{*}\left(\omega_{j}\right)$	0.6102	0.4326	0.3642	0.6976	1.3362	2.5593
$q(\omega_{j})$	0.1017	0.0709	0.1661	0.1163	0.2227	0.4265
*q*(from prices)	1/10	1/10	1/50	1/10	1/5	0.48

**Table 5:** Maxentropic risk neutral option prices (left) from the corresponding ranges (right).

76.752	[69.2, 76.8]
48.645	[44.6, 49.4]
31.349	[29.4, 32.6]
19.392	[19.0, 21.0]
10.070	[09.5, 10.5]
